# Assessing the Risk of Postoperative Complications for Proximal Humeral Fractures Treated by Open Reduction and Internal Fixation in Patients With Diabetes and Chronic Kidney Disease

**DOI:** 10.5435/JAAOSGlobal-D-23-00043

**Published:** 2023-05-10

**Authors:** Jordan Baker, Aaditya Manirajan, Jennifer Lewis, Henry Seidel, Jason Strelzow

**Affiliations:** From the Medical College of Georgia, Augusta University, Augusta, GA (Baker); The University of Chicago Pritzker School of Medicine, Chicago, IL (Manirajan, Ms. Lewis, and Mr. Seidel); and the Department of Orthopaedic Surgery, The University of Chicago Medical Center, Chicago, IL (Dr. Strelzow).

## Abstract

The prevalence of diabetes mellitus (DM) and chronic kidney disease (CKD) has markedly risen over the past three decades. Patients with DM and CKD are at increased risk of infection, immune dysfunction, as well as bone and mineral disorders. Although we know that patients with DM and CKD have these risks, we do not know how these translate to proximal humeral fracture (PHF) healing. We sought to analyze whether these established comorbidities had increased rates of complications after open reduction and internal fixation (ORIF) for PHF treatment.

**Methods:**

Using a national insurance database, 72,365 patients with PHF managed with ORIF were identified using records from 2010 to 2022. Patients were initially split into those with DM and those without and were further stratified by the presence or absence of CKD. For our comparison baseline cohort, patients were not diagnosed with either DM or CKD. Post-ORIF complication rates were assessed looking specifically at nonunion, postoperative infection, and all-cause revision surgery. A logistic regression statistical analysis was also conducted.

**Results:**

Of the 72,365 patients with PHF treated by ORIF, 41,047 were non-DM without CKD (comparison); 17,025 had DM alone (no CKD); 11,729 had DM and CKD; and 2564 had CKD alone (non-DM). Multivariate analysis indicated that patients with DM and/or CKD were at increased risk of developing nonunion (odds ratio [OR] = 1.37, 1.48, 1.23) and all-cause revision surgery (OR = 1.21, 1.11, 1.18) after ORIF for PHF compared with our comparison cohort. In addition, all patients with DM alone (non-CKD) and DM with CKD had an increased risk of postoperative infection (OR = 1.39, 1.26).

**Conclusion:**

The management of PHF is a controversial topic, particularly regarding the degree of intervention and optimal treatment choice. Regardless, using a pragmatic design and reviewing a national insurance database, this study provides information for patients in high-risk populations, specifically patients with DM and CKD, and may prove beneficial when selecting a patient-specific treatment plan. Additional studies are needed to assess varying stages of both DM and CKD in patients who sustain PHF treated by ORIF along with postoperative strategies to minimize complications.

Proximal humeral fractures (PHFs) occur in four anatomical locations of the humerus: the surgical neck, anatomical neck, greater tuberosity, and lesser tuberosity, with the surgical neck being the most common anatomical location for fracture.^1,2^ PHFs have a bimodal distribution, occurring in elderly individuals most commonly with osteoporosis and in younger individuals by high-effect mechanisms, such as impact sports and motor vehicle accidents.^1,2^ Most PHFs can be treated nonsurgically because of the rich vascular supply and cancellous bone surface area.^2^ However, depending on patient factors and injury patterns including the degree of displacement, surgical intervention may be warranted, although no set criteria have been determined and considerable controversy exists around management strategies.^3,4^ Although various surgical procedures can be used to treat PHF, open reduction and internal fixation (ORIF) is the most commonly used.^3,4^ Complication rates following ORIF have been reported to range variably (9.7% to 37%) with revision rates estimated to be between 10% and 12%.^5,6^ Common complications include infection, failure of fixation, screw cutout, and nonunion.^5,6^ Established risk factors of surgical complications include smoking, diabetes mellitus (DM), chronic kidney disease (CKD), and heart disease.^6-8^ Although prior studies have analyzed the various complications after ORIF for PHF in patients with DM and CKD, they have not specifically assessed the rates of nonunion.^9,10^ In addition, using a large national insurance database, correlation can be helpful for future causation studies. National insurance databases provide large trends in complications, such as nonunion, all-cause revision surgery, and postoperative infection, compared with institutional databases that may lack the event rate of these issues to provide unbiased relationships. With a rising rate of DM and CKD, understanding what if any risk these factors pose to fixation is important—particularly as the population ages and the prevalence of these injuries increases.

Elderly patients with PHF often have multiple comorbidities for surgeons to consider. DM and CKD have increased in prevalence over recent decades.^11-13^ In 2013, the incidence of DM was 8.3% worldwide with a projected increase to 10.1% by 2035.^12^ Similarly, as of 2016, CKD affected roughly 11% to 13% of the global population.^13^ More so, 20% to 40% of patients with DM have CKD.^14^ Given the risk of bone and mineral disorder in CKD along with immune dysfunction, risk of infection, and prolonged inflammation in patients with DM, the effect of these comorbidities on surgical outcomes is of interest to orthopaedic surgeons.^15-17^ In patients treated surgically by ORIF, complications include nonunion, all-cause revision surgery, and postoperative infection.^18-20^ Thus, the purpose of this study was (1) to examine the rates of nonunion, all-cause revision surgery, and postoperative infection after ORIF for PHF and (2) to examine whether DM and CKD are risk factors of these complications.

## Methods

### Database

A retrospective review of the national insurance database, Mariner Database, through the PearlDiver Research Database was conducted. At the time of data collection, the Mariner Database included patient records from 2010 to 2022 and contained 157 million patients who were covered with Medicare, Medicaid, self-pay, or commercial insurance. Patient records are searchable with billing codes from the International Classification of Diseases, 9th and 10th Revisions (ICD-9 and ICD-10); the Current Procedural Terminology (CPT); and the National Drug Code. The data in PearlDiver are Health Insurance Portability and Accountability Act–compliant, and this study is exempt from institutional review board review.

### Data Analysis

Patients who sustained PHF were identified in the database using relevant ICD-9-D and ICD-10-D codes (Appendix, http://links.lww.com/JG9/A281). Patients who received ORIF surgical repair were then chosen based on ICD-9-P, ICD-10-P, and CPT codes. Patients were then split into four cohorts: one was a baseline comparison group (non-DM, non-CKD) and the other three included DM without CKD, DM with CKD, and CKD without DM. ICD and CPT codes for DM and CKD were used to query the database and generate each group (Appendix, http://links.lww.com/JG9/A281). A focused review of all subjects for the key complications was performed including rates of nonunion at 6 months, 1-year rates of all-cause revision surgery, and 1-year rates of postsurgical infection (Appendix, http://links.lww.com/JG9/A281). For each complication, we only included them if they occurred within the time frame specified after ORIF (after within 6 months, after within 1 year). For inclusion, patients were required to remain active in the database for a minimum of 1 year. The active period is used to assess patients who remain enrolled with their insurance carrier after a specified period after an event. Patients excluded were either treated nonsurgically or received alternative surgical intervention.

### Statistical Analysis

Descriptive statistics were used to assess the demographics and means. Cohorts were analyzed categorically by univariate and multivariate logistic regression analyses. Only cohorts that displayed *P* ≤ 0.05 in the univariate analysis were incorporated into the multivariate logistic regression analysis. For our multivariate logistic regression analysis, heart disease, tobacco use, and age 60+ years were used to control for confounding variables because they are established risk factors.^7,8^ Heart disease included coronary artery disease, congestive heart failure, and ischemic heart disease. Given the increased prevalence of ORIF in younger patient populations, we considered patients older than 60 years as a separate risk group because they are older and thus less likely to obtain ORIF. Statistical significance was achieved at *P* ≤ 0.05, and odds ratios (ORs) with 95% confidence intervals (CIs) were assessed for each complication. Statistical analysis was computed using R software in the PearlDiver database.

## Results

### Demographics

The database query reported 746,046 patients who sustained a PHF. Seventy-two thousand three hundred sixty-five were treated with ORIF. The total number of patients who did not have DM nor CKD (comparison cohort) was 41,047 (56.7%); 17,025 (23.5%) had DM without CKD; 11,729 (16.2%) had both DM and CKD; and 2564 (3.5%) had CKD without DM (Table 1). Complication rates in the baseline comparison group were 3.4% for nonunion, 8.9% for all-cause revision surgery, and 1.5% for postoperative infection (Table 2). For patients with DM without CKD, complication rates were 4.1% for nonunion, 8.7% for all-cause revision surgery, and 1.9% for postoperative infection (Table 2). Patients with DM and CKD had a nonunion rate of 5.2%, an all-cause revision surgery rate of 9.3%, and a postoperative infection rate of 2.2% (Table 2).In addition, patients with CKD without DM had a nonunion complication rate of 4.3%, an all-cause revision surgery rate of 9.8%, and a postoperative infection rate of 1.5% (Table 2). The average age of patients treated by ORIF for PHFs was 61.8 years (SD = 14.6) (Table 1). Finally, the data were stratified based on sex (male versus Female), which can be referred to in Table 1.

**Table 1:** Demographics of the Study Population

	Female N = 53,635	Male N = 18,730	Total N = 72,365	*P* Value
Age (in yr)				<0.001
Mean (SD)	63.7 (12.6)	54.8 (17.9)	61.8 (14.6)	
Range	0-83	1-83	0-83	
Non-DM without CKD	29,966 (55.9%)	11,081 (59.2%)	41,047 (56.7%)	<0.001
DM without CKD	12,985 (24.2%)	4040 (21.6%)	17,025 (23.5%)	<0.001
Non-DM with CKD	1957 (3.6%)	607 (3.2%)	2564 (3.5%)	<0.001
DM with CKD	8727 (16.3%)	3002 (16.0%)	11,729 (16.2%)	<0.001
Heart disease	20,347 (37.9%)	7157 (38.2%)	27,504 (3.8%)	<0.001
Tobacco use	20,420 (38.1%)	9361 (50.0%)	29,781 (41.2%)	<0.001
Age 60+ years	38,036 (70.9%)	9126 (48.7%)	47,162 (65.2%)	<0.001
Nonunion	1991 (3.7%)	825 (4.4%)	2816 (3.9%)	<0.001
All-cause revision surgery	4571 (8.5%)	1879 (10.0%)	6450 (8.9%)	<0.001
Postoperative infection	722 (1.3%)	486 (2.6%)	1208 (1.7%)	<0.001

CKD = chronic kidney disease, DM = diabetes mellitus

Table 1 demonstrates the demographics of the study population. Average age is included along with separation of male versus female for the groups included in the study.

**Table 2:** Frequency of Complications Between the Comparison Group and Comorbidity Cohorts

Cohort	Total ORIF Patients N = 72,365	Nonunion	All-Cause Revision Surgery	Postoperative Infection
Non-DM without CKD (comparison group)	41,047	1404 (3.4%)	3639 (8.9%)	597 (1.5%)
DM without CKD	17,025	692 (4.1%)^a^	1475 (8.7%)^a^	318 (1.9%)^a^
Non-DM with CKD	2564	111 (4.3%)^a^	251 (9.8%)^a^	38 (1.5%)
DM with CKD	11,729	609 (5.2%)^a^	1085 (9.3%)^a^	255 (2.2%)^a^

CKD =chronic kidney disease, DM = diabetes mellitus, ORIF = open reduction and internal fixation, PHF = proximal humeral fracture

a Indicates *P* < 0.05.

Table 2 displays the frequency of complications after ORIF for PHF in our study population, which includes our comparison group and three comorbidity cohorts. Significant values from a univariate logistic regression analysis are presented in bold and with a star. Non-bolded and starred values are excluded from the multivariate logistic regression analysis.

### Univariate Analysis

Univariate analysis demonstrated that patients with DM alone (non-CKD), patients with DM and CKD, and patients with CKD alone (non-DM) were more likely to develop nonunion and all-cause revision surgery after ORIF for PHF (*P* < 0.001) (Table 2). However, only patients with DM alone (non-CKD) and DM with CKD were more likely to develop postoperative infection after ORIF for PHF (*P* < 0.001) (Table 2).

### Multivariate Analysis

Multivariate analyses demonstrated that compared with our comparison group (non-DM without CKD), patients with DM alone (non-CKD) and patients with DM and CKD were more likely to develop nonunion (OR = 1.37, 1.48; *P* < 0.001, *P* < 0.001), all-cause revision surgery (OR = 1.21, 1.11; *P* < 0.001, *P* < 0.001), and postoperative infection (OR = 1.39, 1.41; *P* < 0.001, *P* 0.001) (Table 3). Patients with CKD alone (non-DM) were also more likely to develop nonunion (OR = 1.23, *P* < 0.001) and all-cause revision surgery (OR = 1.18; *P* < 0.001) (Table 3). Multivariate analysis for postoperative infection in patients with CKD alone (non-DM) was not conducted because the univariate analysis gave an insignificant *P* value.

**Table 3:** Multivariate Logistic Regression Results

Complication Type	Cohort	*P* Value	OR	95% CI
Nonunion	DM without CKD	<0.001	1.37^a^	1.33-1.40
	Non-DM with CKD	<0.001	1.23^a^	1.16-1.31
	DM with CKD	<0.001	1.48^a^	1.43-1.53
	Heart disease	<0.001	1.51^a^	1.47-1.55
	Tobacco use	<0.001	2.04^a^	2.00-2.09
	Age 60+ years	<0.001	0.76^a^	0.75-0.78
	Male	0.05	1.04^a^	1.00-1.08
	Female	<0.001	0.92^a^	0.90-0.95
All-cause revision surgery	DM without CKD	<0.001	1.21^a^	1.20-1.24)
	Non-DM with CKD	<0.001	1.18^a^	1.08-1.30
	DM with CKD	<0.001	1.11^a^	1.08-1.13
	Heart disease	<0.001	1.16^a^	1.14-1.81
	Tobacco use	<0.001	1.70^a^	1.67-1.73
	Age 60+ years	<0.001	0.72^a^	0.70-0.73
	Male	<0.001	1.10^a^	1.08-1.12
	Female	<0.001	0.91^a^	0.89-0.92
Postoperative infection	DM without CKD	<0.001	1.39^a^	1.33-1.44
	DM with CKD	<0.001	1.26^a^	1.20-1.32
	Heart disease	<0.001	1.41^a^	1.36-1.46
	Tobacco use	<0.001	2.03^a^	1.97-2.10
	Age 60+ years	<0.001	0.52^a^	0.51-0.54
	Male	<0.001	1.69^a^	1.63-1.75
	Female	<0.001	0.59^a^	(0.57-0.61

CI = confidence interval, CKD =chronic kidney disease, DM = diabetes mellitus, OR = odds ratio

a Indicates *P* < 0.05.

Table 3 displays the results of a multivariate logistic regression analysis. Four confounding variables (heart disease, tobacco use, age older than 60 years, and sex) were identified and included within the multivariate logistic regression model to control for the effects on the univariate analysis.

## Discussion

Using the PearlDiver National Database, we collected data to quantify the rates of nonunion, all-cause revision surgery, and postoperative infection in patients with DM and CKD who sustained PHF and were treated by ORIF. To our knowledge, there have been minimal data published regarding these risk factors after ORIF for PHF in patients with DM and CKD. Although most PHFs are treated nonsurgically, a proportion may be indicated for surgical management, including high-demand, younger patients or those with severe displacement, vascular injury, brachial plexus injury, and compartment syndrome.^5^ ORIF is currently the mainstay for surgical management of PHF.^5^ A previous study concluded that approximately 6.7% of patients with PHF were treated by ORIF, in comparison with our population, of which 10% of patients with PHF underwent ORIF.^21^ Advances in ORIF locking plate and angular stable technology have improved our tools for fracture fixation and fracture stability, minimizing the risk of screw pullout and fixation failure.^20^ However, there are disadvantages to this procedure, and certain risk factors may pose increased risk of complications.^20^ This includes soft-tissue stripping, fracture at the end of the plate, and poor screw purchase in osteoporotic or comminuted fractures, all of which are risk factors of failure.^20^ Patient-specific circumstances must also be taken into consideration. Even with successful surgical intervention, complications are conceivable, and potential outcomes such as nonunion, all-cause revision surgery, and postoperative infection can be influenced by a variety of factors. Regarding nonunion, proper immune functioning and vascular supply is critical for adequate healing and fusion of fractured bone.^22^ Dysfunction of these factors puts a patient at an increased risk of developing nonunion. Thus, comorbidities, such as DM and CKD, of which macrovascular and microvascular complications can occur along with a proinflammatory state, intrinsically increase the patient's risk of developing nonunion.^22,23^ The goal of this study was to translate this preclinical knowledge into a clinical picture. It is widely known that DM and CKD are risk factors of postsurgical complications for fractures, but quantifying these data are of notable importance for patient education before undergoing these procedures. Assessing comorbidities, patient age, postoperative rehabilitation tolerance, and bone quality are pertinent variables that thoroughly need to be assessed to predict patient outcomes.^19^ Most notably, as displayed in this study, DM and CKD are two comorbidities that can pose notable effects postoperatively.

The pathophysiology of DM and CKD is complex and multifactorial.^11,15^ However, in the context of bone impairment, it is well-known that renal osteodystrophy and a proinflammatory state make this patient population vulnerable to inadequate fracture healing.^11,15^ In CKD, common manifestations include decreased synthesis of vitamin D and erythropoietin in addition to hyperstimulation of the parathyroid gland to compensate for homeostatic dysregulation of calcium.^15^ Consequently, this induces a higher rate of osteoclast activity compared with osteoblast activity.^15^ In addition, in DM, microvascular and macrovascular changes, build-up of advanced glycosylation end-products, and chronic inflammation are known to negatively affect bone architecture and remodeling.^11,24^ In patients with DM, the presence of glycosuria promotes the excretion of calcium in the urine, which is further exacerbated in patients with concurrent renal damage.^11,24^ All these factors affect the integrity of bone remodeling along with adequate oxygen supply systemically.^11,24^ Thus, in the context of PHF in patients with DM and CKD treated by ORIF, our data sought to analyze potential associations between these comorbidities and nonunion, all-cause revision surgery, and postoperative infection. Our findings quantified the increased likelihood patients with DM and CKD have for developing postoperative complications after PHF. Based on the three complications, we focused on for this study nonunion, all-cause revision surgery, and postoperative infection; our data were consistent with our original hypothesis. Overall, based on our three comorbidity cohorts, patients with DM and/or CKD were more likely to develop nonunion, all-cause revision surgery, and postoperative infection compared with our comparison cohort after the ORIF procedure for PHF (Table 3). This aligns with our hypothesis, which was based on the known pathophysiology of both comorbidities previously discussed in this section.

Several studies have evaluated risk factors of failure and complications after ORIF for PHF. There are many studies in the literature that have assessed nonunion, all-cause revision surgery, and postoperative infection in patients receiving surgical treatment by ORIF for PHF. Kavuri et al^20^ looked at nonunion, all-cause revision surgery, and postoperative infection with the frequency of occurrence to be 1.5%, 13.3%, and 1.4%, respectively. However, our study analyzed similar complications while also isolating their incidence to patients with DM and CKD. In addition, comorbidities have been studied to account for other variables involved in perioperative care.^25^ A previous study demonstrated increased lengths of postoperative hospital stays for PHF ORIF procedures in patients with DM compared with those without DM.^26^ This study also concluded that in-hospital complications were 4.0% in men with DM versus 2.9% of men without DM and 2.9% of women with DM versus 1.7% of women without DM.^26^ In addition, when taking patients with DM and CKD into account, risk factors become even more notable. Chen et al^9^ found that patients with DM and CKD had a notable increased risk of postoperative infection with a subdistribution hazard ratio of 1.51 (95% CI, 1.18 to 1.93) after ORIF for PHF. This finding supports the pathophysiology associated with DM and CKD, such as immune system impairment resulting in delayed healing along with infectious risk.^23,24,27^ The positive correlation between postoperative infection and patients with DM and CKD proved to be notable, and we added to this understanding by showing a positive correlation between DM, CKD, and nonunion, which had not previously been done. In addition, as previously mentioned, nonunion is another important postoperative complication that should be considered. A study conducted by Kuo et al^28^ found that patients with CKD had significant increased odds of nonunion after osteosynthesis for intracapsular hip fractures (OR = 5.9, *P* = 0.023). Patients with DM also showed a similar trend in rates of nonunion after surgical management of fractures.^29^ According to Ding et al,^29^ patients with DM showed significant increased rates of nonunion with an OR of 2.11 (95% CI, 1.22 to 3.37, *P* 0.002). Our data showed similar results to these prior studies. However, we specifically looked at PHF, and our data set came from a national insurance database, which provided a markedly larger population for our study. Given the known increased risk of postoperative revision in patients with DM and CKD, we wanted to see whether this translated into our population after ORIF for PHF. This idea was supported by findings in a study which looked at postsurgical complications in patients with DM.^30^ Gougoulias et al^30^ found that patients with DM had a five-fold increase in revision after the ORIF procedure after ankle fracture (OR = 2.30, *P* < 0.001). Our data showed similar results but specifically looked at PHF and used a national insurance database with a larger patient population, which the aforementioned study did not use. Taking all these risk factors into account—postoperative infection, nonunion, and revision—in patients with DM and CKD, our results expanded on similar evidence found in prior studies.

This study does not come without limitations. Using a large data set, we hope that inherent variations of disease are minimized overall. This was a coding study through a large national database, and the results are only as good as the code that is inputted. This study cannot provide cause, but only correlation. As such, DM is a complex disease with a myriad of effects beyond elevated blood glucose and kidney dysfunction. While our study only looked at the general DM population, regardless of the A1C level or length of diagnosis, the progression of DM is insidious, and the longer the disease course without proper management and lifestyle modifications, the higher the risk of developing a myriad of complications.^31^ Like DM, CKD is a progressive disease, and its severity is described through various stages based on glomerular filtration rate.^32,33^ As renal function declines, complications, such as anemia, renal osteodystrophy, electrolyte imbalance, vascular disease, and heart disease, become increasingly prevalent.^32,33^ This study did not look at the various stages of CKD, but rather identified patients who have the disease process itself. In addition, various fracture patterns and degrees of severity affect not only treatment modality but also postoperative outcomes. Although we collected all ICD-9-D and ICD-10-D codes pertaining to PHF, we did not discern the various types of fractures in the multivariate analysis. Despite these limitations, future studies should look at the different stages of progression of DM and CKD and how that affects the complications after the ORIF procedure described in this study. In addition, looking at fracture type and degree of displacement individually could shed light on the complex nature of postoperative complications as displayed in this study.

## Conclusion

Overall, PHF treated with ORIF represents a small proportion of the total population treated for PHF. In this study, we quantified the risks of nonunion, all-cause revision surgery, and postoperative infection in patients with DM and CKD treated by ORIF for PHF. Using a national insurance database, we found that patients with DM and CKD were more likely to experience nonunion, all-cause revision surgery, and postoperative infection after ORIF. These data are useful for orthopaedic surgeons when counseling patients regarding their increased risk of these complications, and it also provides a strong positive correlation between DM, CKD, and complications after ORIF for PHF given the vast past population. Future studies should be able to expand this knowledge by looking at different stages of DM and CKD along with a discerning degree and displacement of fracture to ultimately improve postoperative outcomes.
